# Overview of Biotic Stresses in Pepper (*Capsicum* spp.): Sources of Genetic Resistance, Molecular Breeding and Genomics

**DOI:** 10.3390/ijms21072587

**Published:** 2020-04-08

**Authors:** Mario Parisi, Daniela Alioto, Pasquale Tripodi

**Affiliations:** 1 CREA Research Centre for Vegetable and Ornamental Crops, 84098 Pontecagnano Faiano, Italy; mario.parisi@crea.gov.it; 2 Dipartimento di Agraria, Università degli Studi di Napoli Federico II, 80055 Portici, Naples, Italy; alioto@unina.it

**Keywords:** *Capsicum*, resistance genes, fungal diseases, bacterial spot, viruses, insect, nematodes, QTL

## Abstract

Pepper (*Capsicum* spp.) is one of the major vegetable crops grown worldwide largely appreciated for its economic importance and nutritional value. This crop belongs to the large Solanaceae family, which, among more than 90 genera and 2500 species of flowering plants, includes commercially important vegetables such as tomato and eggplant. The genus includes over 30 species, five of which (*C. annuum*, *C. frutescens*, *C. chinense*, *C. baccatum*, and *C. pubescens*) are domesticated and mainly grown for consumption as food and for non-food purposes (e.g., cosmetics). The main challenges for vegetable crop improvement are linked to the sustainable development of agriculture, food security, the growing consumers’ demand for food. Furthermore, demographic trends and changes to climate require more efficient use of plant genetic resources in breeding programs. Increases in pepper consumption have been observed in the past 20 years, and for maintaining this trend, the development of new resistant and high yielding varieties is demanded. The range of pathogens afflicting peppers is very broad and includes fungi, viruses, bacteria, and insects. In this context, the large number of accessions of domesticated and wild species stored in the world seed banks represents a valuable resource for breeding in order to transfer traits related to resistance mechanisms to various biotic stresses. In the present review, we report comprehensive information on sources of resistance to a broad range of pathogens in pepper, revisiting the classical genetic studies and showing the contribution of genomics for the understanding of the molecular basis of resistance.

## 1. Introduction

Pepper (*Capsicum* spp.) is a fruit vegetable originated in the American tropics and today widely consumed as fresh, dried, or processed products. Around the genus *Capsicum*, there is an increasing interest due to the amazing diversity in plant and fruit characteristics, which make this crop extremely versatile and suitable for innumerable uses. The consumption of pepper has been increased in the last 20 years with a production ranging from 19 to about 40 million tons and a surface area from 2.5 to about 3.8 million of hectares [1]. Further increases are expected due to the greater demand for high-value nutritional products by consumers. Indeed, pepper is a rich source of health-promoting compounds with important nutraceutical and anticancer properties. Despite this favorable trend, several pests and diseases threaten cultivation around the world representing a limiting factor for productivity [2].

The range of pathogens afflicting pepper is very broad and includes fungi (*Phytophthora capsici, Rhizoctonia solani, Verticillium dahliae, Colletotrichum scovillei and truncatum, Leveillula taurica, Fusarium* spp.), bacteria (e.g., *Xanthomonas* spp.), viruses such as *Tospoviruses* (e.g., *Tomato spotted wilt orthotospovirus* and *Impatiens necrotic spot orthotospovirus*), *Potyviruses* (e.g., *Potato virus Y*, *Tobacco etch virus*, *Pepper mottle virus*), *Tobamoviruses* (e.g., *Tobacco mosaic virus*, *Tomato mosaic virus*), *Cucumoviruses* (e.g., *Cucumber mosaic virus*), nematodes (*Meloidogyne* spp.) and insects (e.g., mites, aphids, *Lepidoptera* and thrips). Cultural methods and pesticides are applied to ensure a healthy and profitable pepper crop. Considering the increasing need for sustainable agriculture, the use of resistant plants represents the main strategy to protect pepper cultivation against biotic stresses [2,3,4]. As an example, the limitations imposed in recent years on the use of soil fumigants have led to the growth of interest in the introduction of resistance against soilborne pathogens such as *Phytophthora* spp. and *Meloidogyne* spp. in rootstocks and cultivars [5]. 

In the last decades, most of the pepper breeding programs have been addressed to the development of cultivars or hybrids against a wide range of pathogens and pests. Despite the efforts made, the exploitation of *Capsicum* germplasm (pre-breeding materials, landraces, wild relatives and closed related species) and its use in breeding programs for biotic stress resistance still represent challenging tasks [2]. Indeed, climate changes and the risk of a resistance breakdown, affect the durability of disease resistance, therefore, there is an urgent need to develop new resistant cultivars that can be adapted to varied pedoclimatic conditions. In this frame, gene pyramiding strategies can allow the accumulation of resistance genes in a single genotype and creates more durable and broad-spectrum mechanisms [6]. The strategy can be accomplished combining one or more alleles of major genes [7]. Pyramiding strategies have been successfully used for resistance to pathogens in several crops [8]. 

Some databases are available and refer to a global collection of several materials (wild and domesticated accessions, cultivars, breeding lines, and hybrids) as a source of resistance or tolerance to several pests and diseases. The most important public databases are Chile Variety Database [9], NPGS Germplasm Collection Genebanks from the USDA-ARS [10], World Vegetable Center database [11], The Centre for Genetic Resources, the Netherlands (CGN) of Wageningen University [12], National Bureau of Plant Genetic Resource (India) [13]. The present review aims to provide comprehensive information on the sources of resistance to a broad range of pathogens of pepper, revisiting the classical genetic studies and showing the contribution of genomics for the understanding of the molecular basis of resistance.

## 2. Fungal Diseases

### 2.1. Powdery Mildew

**Figure 1:**
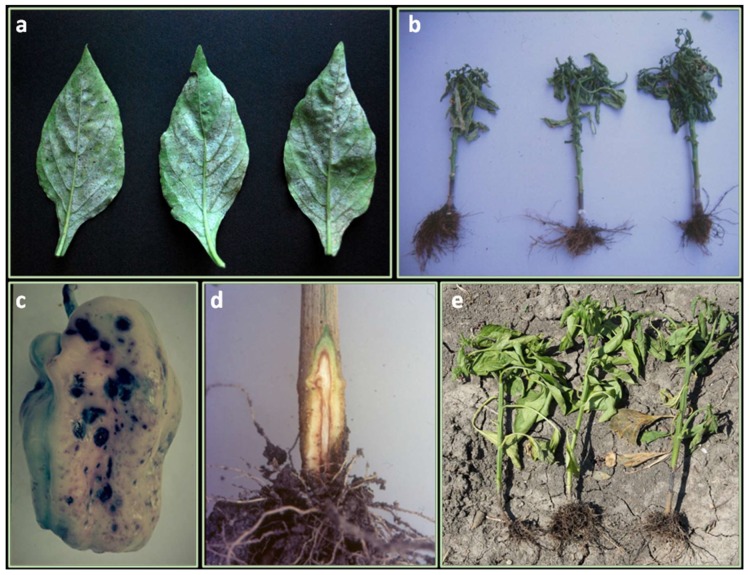
Symptoms and damages caused by fungal disease in leaves, plants and fruits: (**a**) powdery mildew on leaf; (**b**) shriveled plants attacked by *Phytophthora* root rot; (**c**) anthracnose of fruit; (**d**) *Verticillium wilt* with discolored vascular tissue of infected stem; (**e**) Root and stem rot caused by *Rhizoctonia solani*.

The powdery mildew of pepper occurs worldwide and is particularly severe in warm climates, dry or humid, where it causes severe yield losses. The disease, caused by *Leveillula taurica* (asexual stage: *Oidiopsis taurica*), appears as grayish white patches on the undersides of leaves and light green-yellow lesions on the upper leaf surface (Figure 1a). Genotypes from different *Capsicum* species have been reported to be immune or highly resistant to the fungus [14,15,16] (Table 1). 

At least three genes could be involved in the resistance to *L. taurica* in H3 cultivar [17]. The first attempt to map powdery mildew resistance was carried out by Lefebvre and colleagues, which described the quantitative nature of the resistance identifying a robust Quantitative Trait Locus (QTL) on chromosome 6 (*Lt 6.1*), and reported epistatic interactions explaining more than 50% of the genotypic variance [18] (Table 2). A single dominant locus, *PMR1*, located in a syntenic region of 4 Mb of pepper Chr 4 and responsible for the powdery mildew resistance has been reported [19]. Within this locus, two genes across the 622 predicted ones were found to share sequence similarity to the nucleotide-binding site leucine-rich repeat domain containing R proteins (NBS-LRR), which represent clusters of resistance genes in plants. Moreover, the authors identified six molecular markers [one Sequence Characterized Amplified Region (SCAR); five Single Nucleotide Polymorphysms (SNPs)] tightly linked to *PMR1* and useful for marker-assisted selection (MAS) and pyramiding. Phylogenetic analysis based on Genotyping by Sequencing (GBS) data and InDel markers demonstrated a close relatedness of the *PMR1* region from *C. baccatum* and *C. annuum* supporting the introgression of resistance from *C. baccatum*, possibly using *C. chinense* as a bridge species. 

A patent application using the resistant line PBC167 reports a QTL on linkage groups (LG) 1 and 8 explaining 57% of the variance [49]. Using the same line, the QTL was precisely mapped in an interval of ~40 cM on Chr 4 [20].

Functional studies allowed to determine two genes *CaMlo1* and *CaMlo2* as the responsible for pepper resistance to powdery mildew; the loss of function of these genes determine the reduction of disease susceptibility [50].

### 2.2. Phytophthora Root Rot and Foliar Blight

The disease is caused by *Phytophthora capsici,* one of the most destructive pathogens of pepper particularly where the soil is wet, and temperatures remain low (15–23°C) [51]. The oomycete can also cause stem and fruit rot, wilting, stunting, dumping-off, plant death as well as stem and leaf blight (Figure 1b). Separate and independent resistant systems have evolved for each *P. capsici* disease syndrome and independent resistance genes have been detected [52,53]. More than 45 physiological races have been identified within the *Phytophthora* root-rot and foliar blight [54]; for each physiological race, different *R* genes for given disease stages are involved [55]. Therefore, pyramiding multiple genes is essential as it occurs for the *P. infestans* pathosystem in the closely related potato (*S. tuberosum*) [56]. The characterization of pathogen races is traditionally performed utilizing a set of differential hosts which are not always affordable for breeding purposes due to reproduction barriers [57]. Recently, novel races have been identified based on the differential reactions of a set of New Mexico Recombinant Inbred lines (NMRILs) [54]. Identification of pathogen races using NMRILs suggests supplementing the term “race” by the term “virulence phenotype”, to designate the virulence of *P. capsici* isolates to the various host resistance genes [58]. Several *C. annum* accessions resistant to *P. capsici* and carrying a single dominant gene [59], or multiple genes with additive or epistatic effects [60] have been identified (Table 1). Among them, Serrano Criollo de Morelos (CM334) has the highest resistance level to all the disease stages [51]. Host-parasite coevolutionary relationships have been demonstrated [58]. Indeed, both *P. capsici* and resistant germplasm sources have the same geographical origin. Two main chromosomal regions deputed for the resistance to *P. capsici* were identified through a comparative mapping strategy involving three intraspecific *C. annuum* populations [61] (Table 2). From the alignment of the relative genetic maps, a common major QTL was positioned on Chr 5. Afterward, 16 chromosomal intervals containing single or clusters of resistance QTLs for root rot and (or) leaf blight, were identified by using a RIL mapping population [62]. Moreover, the authors reported five QTLs with an effect on the resistance to root rot using an intraspecific F_2_, highlighting the complex polygenic nature of the resistance to *P. capsici*. In the same year, a Random Amplification of Polymorphic DNA (RAPD) marker (OpD04.717) able to amplify a single band in genotypes with high levels of resistance and linked to the major QTL *Phyto.5.2* has been identified [51]. In 2006, Sugita and collaborators detected a major QTL (Phyt-1) on Chr 5 and two minor QTLs on Chr 1 and 11 explaining over 80% and less than 10% of the phenotypic variance, respectively35. *Phyt-1* was in the same chromosomal region of other major QTLs (*Phyto-P*; *Phyt.5.1*; *Phyt.5.2*) identified on Chr 5 previously [51,61,62]. Chromosome 5 was confirmed as the main region involved in the pathogen responses [63]. Kim and colleagues reported seven QTLs, four of which (66.3% of the phenotypic variation) were related to the root rot resistance, while three (45% of variation), were related to dumping off resistance [64]. The authors confirmed the existence on Chr 5 of a major QTL stable across several *P. capsici* populations and isolates [51,61,63,65]. Markers for rapid analysis of resistant genotypes were developed by sequencing the Bacterial Artificial Chromosome (BAC) clones of the Restriction Fragment Length Polymorphism (RFLP) markers closely linked to the major QTLs [64]. An intraspecific RIL (Table 2), was used to map QTLs for resistance against different *P. capsici* isolates in two different studies. The first identified 15 QTLs, seven of which located on Chr 5 explained a phenotypic variation from ~5% to ~50% [66]. The second, allowed to detect 4 QTLs evidencing three main-effect loci related to *P. capsici* resistance. A QTL located on Chr 5 explained over 60% of the heritability of additive effect, being the major-effect gene involved in the pathogen defense response [67]. Using the same RIL population, bulked segregant analysis (BSA) combined with RAPD and Amplified Fragment Length Polymorphism (AFLP) markers allowed to develop a co-dominant SCAR marker (SA133_4) linked to root rot resistance in the region of QTLs contributing to obtain a stable resistance on Chr 5 [66]. Combining BSA and microarray analysis (Affymetrix GeneChips), SNP markers tightly linked to the major QTL on Chr 5 were identified [66,68]. Among them, the marker Phyto5SAR showed the highest logarithm of the odds (LOD) value at the QTL on chromosome 5. Phyto5SAR was in a region containing clusters of resistance genes (NBS-LRR) and a systemic acquired resistance-related gene (*SAR 8.2A*) both associated with plant defense responses. Moreover, within this region, the reliable marker Phyto5NBS1 able to discriminate among susceptible and resistant lines with over 90% accuracy, was designed. A key QTL cluster on chromosome 5 (*Pc5.1*), exhibiting broad-spectrum resistance to *P. capsici* and conferring resistance against at least 12 *P. capsici* isolates worldwide collected was identified [69]. This broad-spectrum QTL showed robust effects in different genetic backgrounds and represented the major target for breeders. Additive and epistatic QTLs have been identified using three isolates of *P. capsici* in 63 F_6_-RILs confirming the Chr 5 as the main region of interest for resistance to root rot [70]. In the same population was identified the gene *CaDMR1* encoding for a homoserine kinase, as the candidate responsible for the major QTL on Chr 5 for resistance to *P. capsici* [71]. Recently, Bulk Segregant Analysis (BSA) combined with Specific locus amplified fragment sequencing (SLAF-seq) allowed to identify *PhR10*: a single dominant gene positioned on the long arm of Chr 10 and responsible for the resistance to race 3 (Byl4) [72]. Functional studies reported that cell death was mediated by the increased reactive oxidative species (ROS) production due to the silencing of the methionine sulfoxide reductase B2 gene (*CaMsrB2*), suggesting that the regulation of pathogen defense responses and oxidative stresses is controlled by ROS accumulation or reduction, respectively [73]. Furthermore, the silencing of *CaRGA2*, a resistance gene analog developed in *C. annuum* CM334 lead to the induction of susceptible disease symptoms after the infection, accompanied by a proliferation of *P. capsici* in pepper tissues [74]. The results of the two studies suggest that gene suppression renders the plants unable to activate the resistance response increasing susceptibility. In 2016, genomic studies allowed to identify over a thousand genes differentially expressed in the resistant line PI201234 among them, 211 were involved in defense responses based on the gene annotations [75]. Validation tests in the resistant Qiemen line, allowed to identify seven genes responsible for many functions related to the prevention of infection (cell wall modification, symptom development, and phytohormone signaling pathways and phytoalexin biosynthesis). The accession PI201234 was used to develop a population of 794 F_2_ individuals by crossing to the susceptible Shanghaiyuan variety [75]. A single dominant gene, *CaPhyto*, on Chr 5 and two candidate genes, *Capana05g000764* and *Capana05g000769*, were found to underly the resistance to race 2. A microsatellite marker (ZL6726) positioned at a distance of 1.5 cM from *CaPhyto*, was validated to be reliable for selecting phenotypes of resistance to the same *P. capsici* race. Several other molecular markers associated with resistance to *P. capsici* have been reported in chile pepper for more rapid selection [51,64,66,68,72,75].

### 2.3. Anthracnose or Ripe Rot of Pepper

Anthracnose causes serious losses of fruits in pre- and post-harvest stages [32,76]. Occasionally, it also damages stem and foliage. The typical fruit symptoms appear as circular water-soaked spots with concentric rings of black acervuli developing beneath the skin (Figure 1c). The spots are often numerous and coalesce, causing softening and rotting of fruits [27]. Anthracnose can be caused by a wide range of *Colletotrichum* species. To date, 24 species infecting pepper have been identified, of which the most common pathogenic are: *C. scovillei* (previously identified as *C. acutatum*), *C. truncatum* (syn. *C. capsici*) and *C. siamense* (previously identified as *C. gloeosporioides*). The latter is less virulent [76,77]. Within these three *Colletotrichum* species, different pathotypes have been identified based on the qualitative and quantitative reaction of fruits at different maturity stages on a set of chilli species and related accessions (Table 1) [27,28]. A major QTL conferring resistance to *C. siamense* and *C. truncatum* and three minor resistant QTLs against *C. siamense* were identified (Table 2) [29]. A single recessive gene conferring resistance to *C. truncatum* was mapped in an interspecific population derived from *C. annuum* cv. Bangchang (susceptible) × *C. chinense* acc. PBC932 (resistant) [30]. The inheritance model was then confirmed in introgression populations [78]. For *C. scovillei*, two major QTLs on Chr 8 and 9, and sixteen with minor effects were detected [79]. Furthermore, five major QTLs located on Chr 5 and conferring resistance to both matured green and matured red fruits, as well as four with minor-effect specific only for the green mature stage have been identified [80]. The first identified sources of resistance in *C. baccatum* (PBC80, PBC81) and *C. chinense* (PBC932) [81], have been extensively used to introgress the resistance in different susceptible *C. annuum* background through conventional breeding and embryo rescue technique [82,83]. PBC80 possesses recessive (*co4*) or dominant (*Co5*) genes located on Chr 12 and Chr 9, respectively. The first was identified in mature green fruit while *Co5* was detected in ripe fruit [84]. PBC932 possesses recessive genes (*co1*, *co2* and *co3*) located on Chr 5 [78]. Two accessions of *C. baccatum* var. *pendulum* (UENF 1718 e UENF 1797) were found very promising to be introduced in breeding programs [31]. Moreover, two SNP maps were constructed from two chilli populations including *C. annuum* Bangchang x *C. chinense* PBC932, and *C. baccatum* PBC80 x CA1316. The validated SNPs are using in anthracnose breeding programs [85]. Recently, sources of resistance to *C. truncatum* and *C. siamense*, under both field and in vitro conditions, have been identified in *C. annuum* accessions (Table 1) [32]. Breeding for resistance to races would broaden the resistance base of chilli cultivars through gene pyramiding of multiple resistance genes [77].

### 2.4. Vascular Diseases

*Verticillium* wilt represents a serious threat to the pepper production worldwide [98]. The disease is mainly caused by the soilborne fungus *Verticillium dahliae* and at a minor extent by *V. alboatrum*. Both pathogens penetrate plants directly or through wounds and spread acropetally through the xylem, causing browning of the vascular tissue, stunting, foliar epinasty, chlorosis and necrosis, wilting and death of the entire plant (Figure 1d). Resistance in peppers is not common in commercial cultivars and is difficult to identify in germplasm sources [99]. Recently, Gurung and colleagues [25], identified eight resistant accessions (Table 1) out of 397 analyzed, of which two (Grif 9073 and PI 439297) conferred resistance also to *Phytophthora* root rot. Although no genetic mapping studies are reported in *Capsicum* for *Verticillium*, molecular markers for assisted breeding have been developed based on the homology with the tomato resistance genes, *Ve* (*Ve1* and *Ve2*) [100]. Like *Ve* gene in tomato, the homolog chilli *CaVe* gene is located on Chr 9 [100] and, through recognition of the *Ave1* effector [101], confers resistance to race 1. Based on the polymorphism between susceptible and resistant accessions in the coding region of *CaVe*, a CAPS marker able to identify *Verticillum* resistant genotypes with the accuracy of 48% was developed [100]. The other vascular disease is caused by *Fusarium*, which determines crop yield losses ranging from 10% to 80% [102]. Several isolates within the *Fusarium* species complex have been linked to pepper wilt. Among them, *F. oxysporum* [103], *F. solani* [33], *F. oxysporum* f. sp. *vasinfectum* [104], *F. redolens* (previously classified as *F. oxysporum* var. *redolens*) [105], and *F. oxysporum* f. sp. *capsici* [106], are the prevailing ones worldwide. *F. verticillioides* (syn. *F. moniliforme*) and *F. pallidoroseum* cause pepper wilting in some parts of India [107]. Maruti and collaborators [33], screening 56 restorer lines and 38 F_1_ hybrids in controlled laboratory conditions, found one genotype (P3) moderately resistant. Moreover, two hybrids, viz., JNA2 × ACB1 × 9608D and Rajaput × P3, showed resistance under sick pot culture conditions. Resistant *C. annuum* genotypes to *F. solani* were also obtained using chemical mutagens such as Ethyl Methane Sulphonate (EMS) [108]. Manu and colleagues [109], studying three crosses viz., SNK x P3, KA2 x P3, and RAJPUT x P3, concluded that the inheritance of *F. solani* resistance was monogenic and dominant. Therefore, heterosis breeding is recommended, to boost the yield in sites where this soil-borne pathogen is widespread. Good sources of resistance to *F. oxysporum*, *F. verticillioides* and *F. pallidoroseum* were found in various *C. annuum* accessions [34,110,111] (Table 1). 

### 2.5. Rhizoctonia Solani

*Rhizoctonia solani* (teleomorph *Thanatephorus cucumeris*) is a destructive soil-borne pathogen that causes several syndromes such as seedling damping-off, root rot, stem rot or canker (Figure 1e) [112]. A wide genetic range of resistance to the most aggressive New Mexican isolate of *R. solani* (PWB-25) was found in accessions belonging to four *Capsicum* species (*C. annuum*, *C. baccatum*, *C. chinense* and *C. frutescens*) [26,37]. In particular, two *C. baccatum* genotypes (PI439410 and PI5556119) were the most resistant to post-emergence inoculation. Nevertheless, the *C. annuum* accessions, Long Chilli (a Korean hybrid) and PI167061, had 67 and 71% resistant individuals, respectively, and could be useful for introducing *R. solani* resistance in *C. annuum* breeding schemes. The investigation of the sources of resistance to *Fusarium* spp., *P. capsici* and *R. solani* was performed in 44 genotypes retrieved from the INIFAP-CEBAJ germplasm bank as well in 141 accessions of *C. annuum* collected in different regions of Mèxico [26]. In total, 26 accessions resistant to *Fusarium* spp., six to *R. solani* and two (BG107 and BG102) to *P. capsici*, were identified. The latters showed mechanisms of resistance to the mixture of all the three pathogens, turning up to be a source of potentially useful genes to be used in breeding programs addressed to the control of wilt diseases.

## 3. Bacterial diseases

### 3.1. Bacterial Spot of Pepper

Bacterial spot is one of the major problems for the cultivation of pepper in tropical and subtropical regions and is principally due to four *Xanthomonas* (hereafter *Xs*.) species: *Xs. euvesicatoria*, *Xs. perforans*, *Xs. gardneri*, and *Xs. Vesicatoria* [38]. 

**Figure 2:**
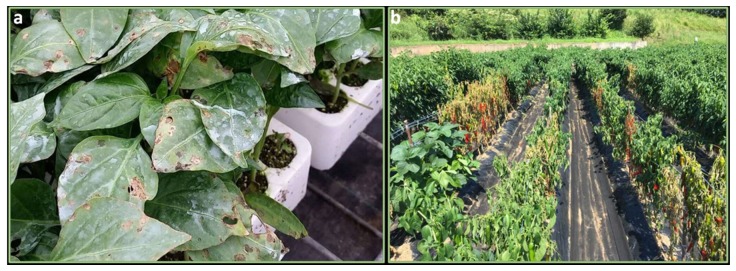
Bacterial diseases in pepper plants: (**a**) bacterial spots on plantlet leaves before transplant; (**b**) extensive wilting in pepper cultivation caused by *Ralstonia* spp.

All parts of the plants are damaged by *Xs*. On the leaves, it causes small, water-soaked, black spots. The spots can coalesce and form large yellow areas that later become necrotic (Figure 2a). On the stem, elongated, raised cankers appear. On green fruits, small, circular, water-soaked, slightly raised lesions are produced. As the disease progresses, spots become brown, roughened, raised with cracked. Yield is reduced because of the scabbed lesions on fruits, which makes fruits unmarketable. The dropping of leaves reduces productivity and exposes fruits to the formation of sunscald.

Nine pepper races (P0-P8) have been identified among *Xs.* strains worldwide [113], and five non-allelic dominant genes (*Bs1*, *Bs2*, *Bs3*, *Bs4,* and *Bs7*) were reported to control hypersensitive reaction to *Xs.* according to the gene-for-gene hypothesis. These genes were found in PI163192 (*Bs1*, *C. annuum*), PI260535 (*Bs2*, *C. chacoense*), PI271322 (*Bs3*, *C. annuum*), PI235047 (*Bs4*, *C. pubescens*) and UNEF1556 (*Bs7*, *C. baccatum* var. *pendulum* [39]. Moreover, two recessive genes (*bs5* and *bs6*), that govern a non-hypersensitive resistance and act additively with each other, were discovered in PI271322, Pep13 and PI163192 (*C. annuum*) [38,40]. One or more of the HR genes have been transferred in near-isogenic lines developed in the Early Calwonder background. Tai and colleagues [114], performed a high-resolution genetic mapping of *Bs2* identifying tightly linked molecular markers in *C. annuum* near-isogenic lines holding introgressions from *C. chacoense* PI260435. A year later, AFLP markers tightly linked to the *Bs3* were identified at a genetic resolution of 0.13 cM [115]. Another marker able to detect a functional nucleotide polymorphism in the *Bs3* promoter (PR-Bs3) was also found by Romer and collaborators [116]. Although *Bs1*, *Bs2* and *Bs3* have been introgressed in several commercial pepper cultivars, mutations in the respective avirulence genes (*avrBs1*, *avrBs2*, *avrBs3*), occurring in the race P6, rendered useless the resistance making this strain highly virulent [117]. It has been demonstrated that the combination of *Bs5* and *Bs6* conferred an additive effect, leading to complete resistance against P6 [117]. Kompetitive Allele-Specific PCR (KASP) genotyping system has been used to develop markers linked to the *Bs3* locus [35]. The developed markers were able to detect susceptible or resistant alleles due to preferential amplification of the transcriptional start site in the promoter region. This approach increased the robustness and throughput of screening resistance loci.

Functional studies evidenced the role of the *C. annuum* peroxidase gene, *CaPO2* in the resistance against *Xs.* [118]. Knock-down of the *CaPO2* gene mediated by virus-induced gene silencing evidenced plants highly susceptible to the *Xs.* infection as well as reduction of hydrogen peroxide (H_2_O_2_) and hypersensitive cell death. On the contrary, overexpression of *CaPO2* exhibited disease resistance, accumulation of H_2_O_2_ accompanied by cell death [118]. These results evidence the role of *CaPO2* in the hypersensitivy mechanism of defence against *Xs.* in pepper.

Moreover, *CaMLO2* has been found to play a role in the *Xs.* resistance. Kim and collaborators [36], demonstrated that the silencing of *CaMLO2* enhanced the resistance against virulent *Xs.*, evidencing the reduced bacterial growth through the boost of reactive oxygen species burst.

### 3.2. Bacterial Wilt

Bacterial wilt (BW) of pepper, is the most devastating soil-borne disease in tropics and in the warmer climates throughout the world [119]. Young plants are rapidly infected and destroyed after the infection (Figure 2b). The older plants first show wilting of the youngest leaves during warm or hot weather day conditions, and after a temporary recovery under cooler temperatures can permanently wither. In the cross-section, plant vascular bundles show a brown discoloration and ooze a white bacterial exudate. Pepper may also show latent infections [120].

BW is caused by *Ralstonia solanacearum*, phylotype I, R. *pseudosolanacearum*, phylotype I and III, and *R. syzyngii* subsp. *indonesiensis* phylotype IV [121]. The three species were previously grouped in *R. solanacearum* species complex (RSSC) and classified into “races” and “biovars” [119,122,123,124]. Virulent isolates were reported in North America and in Japan on pepper cultivars, previously known as resistant [125].

Sources of resistance were found in several cultivated and domesticated pepper accessions (Table 1). 

The inheritance of BW resistance has been established to be controlled by two to five genes with additive effects [45]. The quantitative nature of resistance has been confirmed in studies reporting up to six QTL analysis with additive effects and digenic interactions [87].

A major QTL responsible for resistance to *Ralstonia* was found on Chr 1 (named *Bw1*) [86]. The SSR marker CAMS451 was reported to be tightly associated being mapped in the center of this QTL. Although BW-resistance is thought to be polygenically controlled, the use of this linkage marker may improve the efficiency of breeding BW-resistant cultivars [86].

Recently, the resequencing of the two *C. annuum* cultivars, YCM334 and Tean, allowed to identify novel SNPs and insertions/deletions (Indels) associated with the BW-resistance [46]. The authors detected 10 genes involved in the resistance mechanism including disease resistance proteins, polyprotein, LRR like receptor kinase, N-like protein, CC (coiled-coil)-NBS-LRR, and putative phosphatidylinositol 4-kinase. In 2017, Mou and collaborators identified a further gene, *CaHDZ27*, encoding for a Homeodomain-Leucine Zipper I transcription factors [126], in BW-resistant plants. Gene silencing significantly reduced the resistance down-regulating as well as other defense-related genes (*CaHIR1*, *CaACO1*, *CaPR1*, *CaPR4*, *CaPO2*, and *CaBPR1*). On the contrary, the transient overexpression boosted cell death mediated by the hypersensitive response.

## 4. Viral Diseases

### 4.1. Thrips-Transmitted Viruses

**Figure 3:**
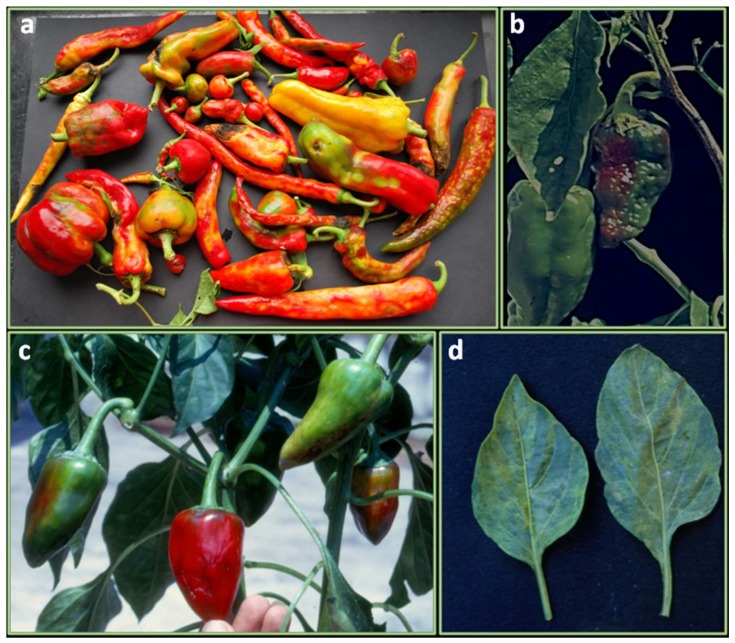
Damages caused by viral diseases in leaves, plants, and fruits: (**a**) extensive spots on fruits caused by *Tomato spotted wilt orthotospovirus* (TSWV) in pepper landraces; (**b**) effect of *Potato virus Y* (PVY) on fruit and stem; (**c**) symptoms on mature fruit caused by *Cucumber mosaic virus* (CMV); (**d**) *Tomato mosaic virus* (ToMV) on leaves.

*Orthotospoviruses* are a group of virus causing serious damages to a wide range of hosts, being transmitted in a circulative propagative manner by at least seven species of thrips (mainly, *Frankliniella occidentalis*). *Tomato spotted wilt orthotospovirus* (TSWV) (Figure 3a), *Impatiens necrotic spot orthotospovirus* (INSV), *Groundnut ringspot orthotospovirus* (GRSV), *Tomato chlorotic spot orthotospovirus* (TCSV), *Watermelon silver mottle orthotospovirus* (WSMoV), *Capsicum chlorosis orthotospovirus* (CaCV), *Groundnut bud necrosis orthotospovirus* (GBNV), *Pepper necrotic spot orthotospovirus* (PNSV), *Pepper chlorotic spot orthotospovirus* (PCSV) were reported to infect *Capsicum* species [127]. Among them, TSWV and INSV are worldwide distributed and represent the only two *orthotospoviruses* occurring in pepper cultivations of Mediterranean area whereas, CaCV, GRSV, and TCSV have emerged as serious pathogens of these crops in India, Australia, Greece (CaCV), Florida (GRSV, TCSV) and South America (TCSV), in more recent years [128,129,130,131,132].

Heritable resistance to TSWV based on a hypersensitive response has been identified in several accessions of *C. chinense*, among them PI152225, PI159234 and PI159236 have been the most adopted in breeding programs [133].

The resistance is due to a single dominant gene (*Tsw*) or a tightly linked group of genes in several *C. chinense* accessions (PI159236, PI152225, CNPH-275 and 7204) [134,135]. The *Tsw* gene has been mapped in the distal portion of chromosome 10 [136]. A CAPS marker (SCAC 568) tightly linked 0.9 cM away to the *Tsw* locus has been identified in a segregant F_2_ population *C. chinense* (PI152225) × *C. frutescens* (PI195301) [137]. The resistance conferred by the *Tsw* gene is overcome by high temperatures (28–33°C) and early plant virus inoculations (two- to four-true-leaf stages) [138]. Recently, the position of *Tsw* has been more precisely assessed in a 295-kb candidate region on chromosome 10 in which NLR genes were clustered [139].

A new resistance inherited as a single dominant gene and indicated either to present a single allele at the *Tsw* locus or to be controlled by a different gene tightly linked to *Tsw*, was found in AC09-207, which is a *C. chinense* accession very similar to PI152225 [140].

Several other *Capsicum* species (*C. frutescens*, *C. chacoense*, *C. pubescens*, *C. galapagoense*, *C. baccatum* var *pendulum* and var *baccatum*), carrying the *Tsw* gene, are known as a good source of resistance too [141,142]. No extensive efforts have been instead carried out to discover the genetic basis of resistance for the other *Orthotospovirus* in pepper. Nowadays the *Tsw* gene is widely used in most commercial pepper hybrids as the unique source of TSWV resistance [143], however, its extensive adoption has triggered the rapid emergence of resistant-breaking (RB) isolates soon after their introduction. To date, reports on *Tsw*-resistance breakdown are from Italy, Spain, Australia, Hungary, Turkey, Argentina, and more recently in China and California [144,145,146].

Mixed infections with RB-TSWV and wild-type TSWV (WT-TSWV) isolates are very frequent in pepper cultivations. The co-infection by WT and RB isolates induces synergism effects with the appearance of necrosis on the apical leaves of TSWV-resistant genotypes [147].

For these reasons, the search for sources of resistance and/or tolerance (reduction of severity symptoms) to RB-TSWV strains in wild or exotic germplasm is essential to develop new varieties. Studies on *Capsicum* germplasm resistant to RB-TSWV are currently in progress in different countries [148,149,150]. A good level of tolerance to WT- and RB-TSWV isolates in the *C. baccatum* accession PIM26-1 [150]. 

Regarding CaCV, sources of resistance were found in *C. chinense* PI90972 [151]. A transcriptome analysis and expression profiling of CaCV evidenced about 2500 genes differentially expressed between susceptible and resistant genotypes with different functions (pathogenesis, cell death, and hormone-mediated signaling pathways and enzymes for defense-related pathways) [152]. Genes involved in localized cell death, cell signaling, synthesis of antimicrobial compounds and PR proteins were found highly upregulated. Moreover, two resistance NB-LRR candidates were putatively involved in a CaCV-resistant breeding line carrying introgressions from *C. chinense*.

### 4.2. Aphid-Transmitted Viruses

#### 4.2.1. Potyviruses

Potyviruses likely represent the most spread viruses infecting peppers involving aphids as vectors of transmission [153] (Figure 3b). Seed transmission could occur, although, this has not been conclusively demonstrated in *Capsicum* [127,129]. Pepper can be infected by at least eleven different potyvirus species [127]. Among them, *Potato virus Y* (PVY) is worldwide distributed and is the only one severely affecting pepper crops in Europe [154]. PVY exists as three pathotypes (PVY-0, PVY-1, and PVY-1,2) according to the expressed virulence [153]. The other potyviruses infecting pepper have a narrowed geographical distribution. Therefore, many of them such as TEV, PepMoV, *Pepper severe mosaic virus* (PepSMV), *Pepper yellow mosaic virus* (PepYMV), *Perù tomato mosaic virus* (PTV), and the tentative species, *Ecuadorian rocoto virus* (EcRV) are distributed in South America and have been detected sporadically in other continents, while, *Chilli veinal mottle virus* (ChiVMV), *Chilli ringspot virus* (ChiRSV) and recently PepMoV are present in Asia, while Pepper veinal mottle virus (PVMV) is confined in Africa [127,154].

Several resistance genes to potyvirus are reported in pepper. The *pvr1* locus, showing various alleles with different resistance levels to TEV (*pvr1*, *pvr1^2^*), PVY-0 (*pvr1*, *pvr1^1^*, *pvr1^2^*) and PepMoV (*pvr1*), was identified in *C. chinense* PI159236 and PI152225 [155,156], while the *pvr1^1^* was detected in *C. annuum* cv. Avelar, Yolo Y, CM334, PI264281, and *C. frutescens* I5491 [141,156]. The *pvr2* resistance alleles (*pvr2^1^*, *pvr2^2^*, *pvr2^3^*) are effective against PVY-0, PVY-1, TEV (common strain), and are found in the *C. annuum* accessions Yolo Y, PI264281, SC46252, Florida VR2135. The allele *pvr2^1^* (Yolo Y) is effective only against PVY-0, while *pvr2^2^* (Florida VR2) is effective against PVY-0, PVY-0,1, and TEV. The allele *pvr2^3^* (Perennial) confers partial resistance to PVY. Mapping results showed that these genes were organized in a cluster of recessive genes on Chr 4.

Based on the co-segregation analysis, *pvr2* was found to corresponds to the eukaryotic translation initiation factor 4E *(eIF4E*) [157]. The recessive resistance was probably related to the incompatibility between the potyvirus genome-linked protein (VPg) and *eIF4E* which occurred in resistant genotypes. Based on homology to *eIF4E* and allelism tests between *pvr1* and *pvr22* (both mapping in the same genetic locus of Chr 3) it has been suggested a nomenclature re-designation of *pvr2^1^* and *pvr2^2^* in *pvr1^1^* and *pvr1^2^*, respectively [158]. Mutations in the *eIF4E* and *eIF(iso)4E* genes in pepper were identified through a cDNA eco-tilling platform within 233 cultivated accessions of Capsicum [159]. The authors reported five new *eIF4E* variants (named as *pvr2^10^*, *pvr2^11^*, *pvr2^12^*, *pvr2^13^*, and *pvr2^14^*) related to PVY-resistance responses which represent an excellent allele reserve against the changing nature of viruses, to use in breeding programs. 

The *pvr2* alleles, *pvr2^1^* and *pvr2^2^*, have been used extensively to breed potyvirus resistant pepper cultivars for more than 50 years. Both alleles confer efficient resistance toward PVY, while the only *pvr2^2^* is effective against TEV. The resistance of *pvr22* proved extremely durable against PVY. To date, some *pvr2^1^* and *pvr2^2^*-breaking isolates have been described [160]. However, they are not very prevalent so the cultivars carrying the *pvr2^1^* and *pvr2^2^* resistance continue to be used in breeding programs. 

The *pvr3* gene was reported in *C. annuum* cv. Avelar and confers resistance to PepMoV141. The pvr4 gene derived from *C. annuum* CM334 confers resistance to PVY-0, PVY-1,2, and PepMoV142. Other sources of this gene were found in *C. chinense, C. frutescens, C. baccatum* var. *pendulum*, *C. praetermissum* and *C. galapagoense* accessions using the CAPS marker named CSO [141,161]. 

The recessive loci *pvr5* and *pvr8* from *C. annuum* CM334 provide resistance to PVY-0 and PVY-1 isolate P-62-81, respectively [162]. 

Pepper plants expressing the pvr6 gene from *C. annuum* cv. Perennial, mapped on Ch 3, are resistant to ChiVMV145. The dominant gene *Pvr7* from *C. chinense* PI159236 confers resistance to PepMoV Florida (V1182) strain and is tightly linked to Pvr4146. *Pvr4* and *Pvr7* are mapped on Chr 10 tightly linked to *Tsw*, which confers resistance to TSWV116. Therefore, this chromosome is considered a main cluster of dominant resistance genes in pepper. Venkatesh and collaborators [163], demonstrated that the dominant PepMoV resistance in *C. annuum* cv. 9093 could be derived from *C. annuum* CM334, and that *Pvr4* and *Pvr7* loci should be considered as the same locus.

Moreover, dominant allele *Pvr4* confers a wide range of resistance against several potyviruses (PVY, PepMoV, PTV, PepSMV, and PepYMV) [154].

QTLs involved in the complete and partial resistance to some PVY isolates (To72 and Son41) were identified in eleven chromosomal regions, near *pvr2* and *pvr6* (Table 2) [164]. These QTLs reduce PVY symptom intensity and improve greatly the durability of the major-effect gene pvr23, which alone can be rapidly broken down [165]. Four additional major QTLs explaining over 70% of the variation with additive and epistatic interaction were identified [89]. The authors showed how the resistance breakdown frequency for *pvr2^3^* was under the control of three main QTLs, suggesting a pleiotropic effect on the durability of the major resistance gene.

Different markers have been developed for resistance-assisted breeding to potyviruses. A CAPS marker tightly linked to *Pvr4* was developed by BSA-AFLP [161]. A SCAR marker (SCUBC19_1423_) linked to *Pvr4* was instead developed by BSA-RAPD [166]. Both markers were mapped on Chr 10 at distance variable from 5 to 10 cM and can be used for routine selection of PVY resistant lines.

Three allele-specific CAPS markers able to detect three recessive viral resistance alleles *pvr1*, *pvr1^1^*, and *pvr1^2^* and a functional SNP marker at the *pvr2-eIF4E* locus, have been developed [156,167]. The use of the four primers in a single PCR experiment, allow differentiating alleles in homozygous and heterozygous genotypes. Through KASP-PCR, it was possible to develop a marker in the coding region for the cloned *pvr1* resistance gene [35]. The KASP_pvr1 was validated using a *C. chinense* F_2_ population derived from Habanero (*pvr1+*/*pvr1+*) x PI159234 (*pvr1*/*pvr1*) [158]. The genetic factors underlying the number of PVY particles entering the plant and the accumulation at the systemic level have been studied using a genome-wide association study (GWAS) approach in a collection of ~260 *C. annuum* accessions [168]. Among the over 10 thousand SNPs identified through GBS, seven were highly associated with the resistance being located on chromosomes 4, 6, 9 and 12. Two of them on Chr 4 were closely linked to *pvr2* in the region encoding the *eIF4E*, whereas, the SNPs detected on Chr 6 and 12 colocalized with previously reported QTLs.

Investigations toward the dissection of the genetic basis of ChiVMV has also been carried out reporting novel codominant markers for ChiVMV [169]. One CAPS marker tightly linked to the ChiVMV resistance locus and two high resolution melting (HRM) markers were developed through BSA-AFLP and mapped on Chr 6. Next-generation sequencing (NGS) has been also used to generate molecular markers tightly linked to *Pvr4*. Over 5000 single nucleotides variances in the NB-LRR gene regions were identified and converted into PCR-based markers [170]. More recently, the *Cvr1* gene has been mapped to the short arm of Chr 6 of the resistant variety CV3 [171]. The region was reported to cluster several other NLR genes involved in resistance mechanisms. Furthermore, the authors identified SNP markers useful for assisted breeding of ChiVMV and for the fine mapping of resistance genes.

#### 4.2.2. Cucumoviruses

*Cucumber mosaic virus* (CMV), is the main representative of *Cucumovirus* and is transmitted mainly by *Myzus persicae* and *Aphis gossypii*. CMV reduces quality and fruit yields (Figure 3c), especially in the early infections; yield losses greatly can reach 80% [172]. CMV can occur in nature in mixed infection with other viruses with synergistic effects, i.e., CMV and PepMoV [173]. Furthermore, the coinfection with CMV can reduce plant resistance against other viruses as in PepMoV and ChiVMV resistant pepper plants [174]. 

CMV isolates are classified in subgroups I (clade A and B) and II. Isolates of subgroup I, clade IA, and subgroup II are distributed worldwide while most of the isolates of clade IB are from East Asia. Pepper is more frequently affected by CMV isolates of subgroup I. 

A single dominant resistance gene against CMV (*Cmr1*), identified from the *C. annuum* cv Bukang, was located in the centromeric region of pepper Chr 2. It inhibits the systemic movement of CMV isolates of subgroup IA [175]. A new isolate of CMV belonging to subgroup IB and designated as CMV-P1, has emerged in Korea and is able to break down the resistance conferred by *Cmr1* [176]. Recently, a new single recessive gene, *cmr2*, able to confer resistance to CMV-P1 has been identified using a combining BSA and allelism tests [177]. BSA allowed detecting a single AFLP marker located at 16 cM from *cmr2*. The analysis has been corroborated by inheritance and allelism tests in segregating populations developed using as a source of resistance Lam32 (an Indian *C. annuum* cultivar carrying the *cmr2* gene). This novel gene provides a broad spectrum of resistance to several CMV strains including the common CMV_Korean_ and CMV_FNY_.

Almost all the CMV resistance sources identified in *Capsicum* spp. (Table 3) display a partial resistance controlled by multiple genes [177]. The resistance reported in *C. annuum* Perennial is due to various mechanisms [178]: partial resistance to initial virus infection [88], inhibition of virus multiplication [179], and inhibition of long-distance movement of the virus [180]. The resistance in *C. frutescens* BG2814-6 is instead expressed at the level of replication and cell-to-cell movement [181]. Several of these are ontogenetic depending on the pepper developmental stage [182]. These resistance mechanisms restrict only partially the virus translocation within plants but confer a good level of protection in the field, particularly when different sources were combined into a cultivar [179].

Three chromosomal regions on Chr 3, 11 and 12 with additive or epistatic effects involved in resistance to the CMV systemic movement and explaining 57% of the phenotypic variation (Table 2) were reported [164]. In addition, four QTLs significantly associated with resistance to CMV and a major QTL with digenic interaction on Chr 11 associated with genes conferring resistance to TMV were identified [90]. This QTL was confirmed by Caranta and colleagues [91], which reported the existence of four additive and two epistatic QTLs, as well as of a major QTL on Chr 12 (*cmv 12.1*) explaining between the 45% and 63.6% of the phenotypic variation [91]. Two major QTLs on Chr 5 and 11 explaining a total of 55% of the total phenotypic variation associated with the tolerance to CMV_HB-jz_ strain were further identified [92].

Recently, NGS has been used to identify novel genomic regions underlying CMV resistance. By means of GBS, two novel major QTLs responsible for the resistance to CMV-P1 were identified [93]. The two QTLs were positioned on the Chr 5 (52.7–58.1 cM) and 10 (21.9-32.5 cM) and explained about 20% of the phenotypic variation, respectively. Using SLAF-seq a single gene located on Chr 2 (*CA02g19570*) was reported to be the candidate for the QTL *qCmr2.1* conferring resistance to CMV_FNY_ [94]. Furthermore, a major QTL on Chr 11 was identified. By means of the same genomic strategy, it was possible to detect three additional QTLs for resistance to the CMV_HB-jz_ strain [95]. The major QTL, explaining about 20% of the phenotypic variation, was identified on Chr 11 confirming the importance of this chromosomal region for resistance to CMV. Besides the identification of QTLs, SLAF-seq has allowed the development of functional markers linked to CMV-resistant to be used for MAS in pepper.

### 4.3. Whitefly-Transmitted Viruses

Viruses belonging to the genera *Begomovirus* and *Crinivirus* are transmitted by different species of whiteflies, representing a danger for the cultivation of pepper in different World regions.

#### 4.3.1. Begomoviruses

The genus *Begomovirus* contains viruses transmitted by the whitefly *Bemisia tabaci* persistently. At least 37 ratified and 6 candidate species have been described as naturally infecting pepper. Many of them cause serious diseases in pepper crops in Asia and America [127]. The diseases caused by *Begomoviruses* are easily recognized by their distinctive symptoms ascribed to three types: a) vein yellowing; b) yellow mosaic and c) leaf curl. 

Among *Begomoviruses*, *Chilli leaf curl virus* (ChiLCV) is one of the most destructive disease for chilli pepper. The virus is distributed in almost all equatorial regions of the World [183]. *Pepper golden mosaic virus* (PepGMV) (previously named Serrano golden mosaic begomovirus and *Texas pepper begomovirus*) and *Pepper huastego yellow vein virus* (PHYVV) represent a new threat for pepper production in Central America. *Pepper leaf curl virus* (PepLCV) has been reported in India, United States, Nigeria and several other countries such as Pakistan, Bangladesh, and Indonesia [184]. *Tomato yellow leaf curl virus* (TYLCV) is one of the most devastating plant viruses of tomato whereas in other crops such as cucurbits and peppers is asymptomatic [185]. The virus has been reported on pepper crops in some areas of the Mediterranean basin [186,187].

The begomovirus, *Tomato leaf curl New Delhi virus* (ToLCNDV), represents an important constraint to tomato production, in the Indian sub-continent. In recent years the virus has been rapidly spreading into several countries of the Mediterranean basin causing significant economic losses on cucurbit and tomato [188]. Recently, it has been recovered in Italy in pepper plants showing yellowing and leaf curling [189]. 

Synergistic interactions between different begomoviruses infecting pepper can cause the breakdown of natural resistance in the host plant [190]. 

Despite the increasingly devastating effect of begomoviruses of pepper in many areas of Asia, Central America, and West Africa, breeding programs have not yet produced resistant commercial varieties due to the genetic nature of resistance, which is governed by major recessive genes [191]. The extent in the identification of resistant germplasm and of markers linked to minor genes were done for the ChiLCV-VNS (Varanasi isolate) strain [191].

With respect to PepLCV, an inheritance study of resistance using the partially compatible interspecific cross (PBC-535 X Bhut Jolokia), revealed the monogenic recessive nature [192]. Transcriptomic analysis evidenced 234 unique genes up-regulated in resistant genotype BS-35 respect the susceptible IVPBC535 indicating that gene expression in the resistant genotype responded strongly to PepLCV [193].

Recently, the analysis of 100 *Capsicum* spp. accessions in two locations of Thailand, allowed to identify the accession PP99 as the main source of resistance [194]. The other four genotypes (PP1037-7644-1, PBC148, PBC149, PBC502, PBC518, and PBC601) were classified as highly resistant at both locations. In any case, no accession was identified as being immune to the disease. 

There are several reports of resistance sources to PHYVV in *Capsicum*. Trujillo-Aguirre and Díaz-Plaza [195], found genetic resistance to PHYVV and PepGMV in wild populations of *C. chinense* from Southeast Mexico. Hernández-Verdugo and colleagues [196], found genetic resistance to PHYVV in wild populations of *Capsicum* from Northwest Mexico. More recently, Retes-Manjarrez and collaborators [197], reported the UAS12 line (*C. annuum*) as the most promising genetic resource for its high resistance conferred by at least two genes. 

Resistance to PepGMV in BG-3821 accession (*C. chinense*) is probably controlled by two genes with either additive or duplicate recessive epistatic action [198]. Moreover, the author indicated that the resistance is associated with reduced virus replication and movement, and the induction of genes associated with systemic acquired resistance (SAR).

#### 4.3.2. Crinivirus

*Tomato chlorosis virus* (ToCV) is emerging as a problem worldwide resulting in severe damage, especially to tomato crops [199]. This virus is transmitted in a semipersistent manner by the whitefly species *Bemisia tabaci*, *Trialeurodes abutiloneus* and *T. vaporariorum* [200]. Although tomato is the main crop affected by this crinivirus, the virus has been also reported on sweet pepper plants in greenhouses of southern Spain, Brazil, Costa Rica, Tunisia, and Saudi Arabia [200]. Stunting accompanied by curling, interveinal yellowing and abnormal elongation of leaves, reduced fruit number and size are characteristic of ToCV infections in pepper. No information on sources of resistance to ToCV has been reported in *Capsicum* germplasm, to date.

### 4.4. Viruses Transmitted by Contact

#### Tobamoviruses

*Tobamoviruses* are mechanically transmitted and represent the most damaging viruses for pepper in protected cultivations [127]. The most prevalent in pepper are *Tobacco mosaic virus* (TMV), *Tomato mosaic virus* (ToMV) (Figure 3d), *Bell pepper mottle virus* (BPeMV), *Pepper mild mottle virus* (PMMoV), *Paprika mild mottle virus* (PaMMV), *Obuda pepper virus* (OBPV), *Tobacco mild green mosaic virus* (TMGMV)109. These viruses are particularly stable and for this reason, they remain infectious in contaminated plant residues, compost, soil, and irrigation water. They are easily transmitted by contact and seeds. Seeds can be externally or more rarely internally (endosperm) infected [211]. *Tobamoviruses* infecting *Capsicum* plants are classified into four pathotypes, P_0_ (TMV and ToMV), P_1_ (PaMMV), P_1.2_ and P_1.2.3_ (PMMoV), based on the reaction of pepper cultivars carrying different L resistance genes (*L^1^*, *L^2^*, *L^3^*, and *L^4^*) [212]. *L^1^* confers resistance to P_0_ strains; *L^2^* confers resistance to P_0_ and P_1_, L_3_ confers resistance to P_0_, P_1_ and P_1,2_, *L^4^* confers resistance to all strains (P_0_, P_1_, P_1,2_ and P_1,2,3_) [213]. Studies have identified the viral coat proteins (CPs) as elicitors of *L* genes-mediated resistance [214,215] and amino acid changes responsible for overcoming *L^3^* and *L^4^*-gene-mediated resistance in the CP [215,216,217]. The *L* locus was mapped to the sub-telomeric region of pepper Chr 11, 4.0 cM apart from the RFLP marker TG36 [218]. This region was syntenic to the tomato Chr 11 which carries the *I2* resistance genes for *F. oxysporum* [219]. *L^1^* was mapped in *C. annuum* to Chr 11 through an integrated molecular linkage map of cultivated pepper (*C. annuum*) obtained from the alignment of three DH (double haploids) maps [218]. *L^4^* from *C. chacoense* was mapped by Matsunaga and collaborators [220] and confers resistance to the most aggressive and common tobamovirus pathotypes P_1.2.3_ [221]. Good sources of resistance to pathotypes P_1.2.3_ were recently found in several accession of *C. baccatum* var. *pendulum* and in germplasm belonging to *C. pubescens*, *C. frutescens*, *C. chinense* and *C. praetermissum* using the dominant marker 060I2END linked to the *L^4^* locus [141]. 

In addition to these classical *L* genes, another *Tobamovirus* resistance gene, *L^1a^*, has been identified [210]. The authors demonstrated that in contrast to *L^1^*, the gene *L^1a^* mediates resistance to P_0_ pathotype (TMV and ToMV), independently by the temperature, and to P_1_ (PaMMV) at 24 °C. A year later, the same research group, identified a single incompletely dominant gene different from the *L* gene designated as *Hk*, which confers resistance to P_1_ pathotype (PaMMV) at 30 °C but not at 24 °C. The source of resistance *C. annuum* cv Nanbu–Ohnaga, although resistant to PaMMV was ineffective against any of the other *Tobamovirus* pathotypes (TMV P_0_ and P_1_, and PaMMV P_1,2_) [210]. The P_1.2.3.4_ pathotype of PMMoV, which differs from P_1,2_ for two amino acids in the coat protein, can break the *L^4^* resistance, indicating the need to identify *R* genes effective against this virus strain [217]. Efforts to develop molecular markers linked to *L* genes are reported, such as the SCAR marker WA31-1500S linked 1.5 cM to *L^4^* and able to distinguish resistant from susceptible accessions [220]. 

The *L^3^* resistance gene of *C. chinense* was positioned in a 400-kb region of pepper Chr 11 containing clusters of R-like genes and highly repetitive sequences, confirming, the presence of many repetitive sequences of the *L* locus [222,223]. Several tightly linked markers, including the 189D23M located within 0.1 cM of the *L^3^* gene, were identified. However, inconsistencies in the genetic distances of these markers from the *L^3^* locus [222], suggested linkage disequilibrium in the underlying region containing the *L^3^* locus. Via comparative analysis, Yang and collaborators [221], developed *L*-linked markers using the BAC sequence information corresponding to the syntenic tomato *I2* (conferring resistance to *F. oxysporum* f. sp. *lycopersici*) and potato *R3* (conferring resistance to *P. infestans*) loci, three of which (087H3T7, 060I2END and 158K24) were found to be in linkage to the L3 and L4 loci. Further mapping analysis demonstrated a different linkage of the previously identified 189D23M to *L^4^* respect *L^3^*, suggesting the possible existence of different genes closely linked instead that different alleles at the same locus. Three years later, the same research group [224], developed a marker (L4segF&R) located within 0.3 cM from *L^4^* using diverse segregating populations and breeding lines. Given its not complete co-segregation with the *L^4^* gene, the marker is considered as a candidate of resistance not- *L^4^* related. Furthermore, several allele-specific markers for the *L* locus were developed using the LRR-encoding domain of the NBS-LRR disease resistance gene candidate for the different L alleles. 

Functional studies reported different transcription factors involved in the infection of Tobamovirus. The *CaWRKYb* gene of the WRKY family was reported to be rapidly induced during TMV (pathotype P_0_) infection in hot pepper [225]. A *CaWRKYb*-knockdown evidenced a reduced resistance level in plants as a result of minor hypersensitive response upon TMV-P_0_ infection. The compromised resistance to TMV-P_0_ was due to major TMV accumulation through decreased expression of pathogenesis-related genes of *C. annuum* (*CaPR-1*, *CaPR-5* and *CaPR-10*). The results suggested that *CaWRKYb* plays as a positive role in defense-related signal transduction pathways in hot pepper [225]. The gene *CaWRKYd*, isolated from microarray analysis in TMV-P_0_-inoculated hot pepper (*C. annuum*) plants is a new transcription factor that belongs with a subgroup (IIa) of the WRKY family [226]. *CaWRKYd* transcripts were reported to be induced by P_0_ inoculation and hormone treatments [226]. The silencing of this gene affected TMV-P_0_-mediated HR cell death and the accumulation of TMV-P_0_ coat protein in local and systemic leaves. Moreover, a reduction of expression of some pathogenesis-related (PR) and HR (hypersensitivity response)-related genes was evidenced after silencing, confirming that this gene modulates HR cell death by regulating downstream gene expression. The same year, Huh and collaborators [227], analyzed the function of *C. annuum* basic transcription factor 3 (*CaBtf3*) of the NAC family through VIGS and found its involvement in HR cell death related to TMV-P_0_ infection.

### 4.5. Pollen Transmitted Viruses

#### Ilarviruses

*Ilarviruses* are transmitted mechanically by thrips feeding on pollen grains containing the virus or by carrying pollen grains contaminated by the virus. *Tobacco streak virus* (TSV), is the main species including a wide host range, with at least 200 susceptible species. TSV was reported causing systemic necrosis, dark streaks on stems and petioles and tip necrosis on pepper in Argentina and in India [228,229].

*Parietaria mottle virus* (PMoV), was identified on bell pepper in Southeast Spain [230], and on pepper ecotypes and commercial hybrids in Southern Italy [231]. Infected plants showed rings, mosaic and necrotic patches of the leaves, necrotic stems, and brown patches and corky rings on fruits [231]. No source of genetic resistance has been investigated in *Capsicum* spp., to date.

## 5. Arthropods and Nematode Pests

In plants, insects and arthropods exert their activity destroying tissues, causing energy stresses and competing for nutrients. Furthermore, insects are key vectors of several pathogens. In pepper, more than 21 insect and non-insect pests cause heavy yield losses worldwide [232]. A strategy to reduce pest damages and minimize the use of insecticide applications is the adoption of pest-resistant genotypes. Unfortunately, studies on plant genotypic variation in resistance to arthropods and pests in the genus *Capsicum* are still scarce to date and resistant commercial varieties (or rootstocks) are available only for root–knot nematodes.

### 5.1. Thrips

**Figure 4:**
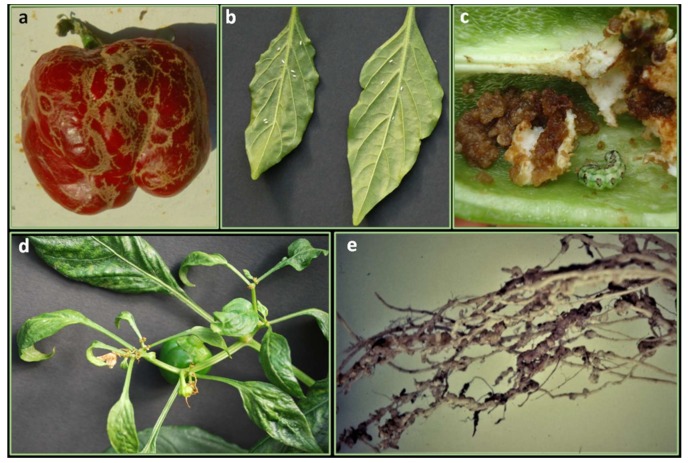
Arthropods and nematodes pests: (**a**) stripes on fruits caused by thrips feeding (*Frankliniella occidentalis*); (**b**) adult stages of whiteflies (*Bemisia Tabaci*) on the underside of leaves; (**c**) damages on fruit caused by of cotton bollworm larvae (*Helicoverpa armigera*); (**d**) distorted leaves and damages on inflorescences caused by broad mite feeding (*Polyphagotarsonemus latus*); (**e**) galls or “knots” on pepper roots caused by nematode (*Meloydogyne* spp.) feeding.

Thrips (Thysanoptera: Thripidae) cause damages directly by feeding on leaves, fruits or flowers, and indirectly by transferring viruses, especially TSWV in pepper worldwide. There are at least 16 species of thrips that attack *Capsicum* [233]. Among them, *F. occidentalis* (Figure 4a), is the major species found on pepper in Europe [234], and in Asia as well [235]. Several pepper accessions have been found to carry resistance to thrips which may be exploited further to breed resistant varieties increasing the effectiveness of thrips control and delay or reduce the transmission of viruses [236,237]. Six *C. annuum* and *C. baccatum* accessions (Table 4) were identified as good sources for resistance against *Thrips parvispinus* and *F. occidentalis* [96]. These studies also confirmed the good level of resistance of two accessions: Keystone Resistant Giant and CPRO-1 [237,238]. The latter showed a reducing of thrips reproduction. Moreover, the leaf-based resistance to F. occidentalis and *T. tabaci* have been demonstrated species-specific, being not correlated [239].

An attempt to identify chromosomal regions responsible for resistance has been reported [96]. The authors developed a genetic map in an F_2_ population derived from the cross between *C. annuum* AC 1979 (female parent, susceptible) x *C. chinense* 4661 (male parent, resistant). A single QTL explaining about 50% of the genetic variation was detected for three traits of resistance (damage caused by larvae and the survival of first and second instar larval stages), all co-localized near the same marker on Chr 6. Resistance parameters and trichomes density were not correlated suggesting that the latter don’t exert major effects on resistance mechanisms to thrips.

### 5.2. Tobacco Whitefly 

*Bemisia tabaci* (*Hemiptera: Aleyrodidae*) (Figure 4b) has become a serious threat to crop production not only by causing direct feeding damages but also being a vector capable of transmitting efficiently more than 200 plant viruses, 90% of them Begomoviruses [240]. The frequent use of pesticides leads to resistant whiteflies so that the use of resistant varieties, biological control or a combination of them is strongly recommended [241]. Sources of resistance were found surveying 44 *Capsicum* accessions under both screen-house (Wageningen, Holland) and in-field test conditions (tropical area in Indonesia) [242] (Table 4). A strong antixenotic and antibiosis effect against *B. tabaci* was found in P2, P4, ACC1 and ACC12 accessions of spp. (Table 3) [243]. An antibiosis mechanism was also suggested for the whitefly-resistant accessions IAC-1544 (*C. frutescens*), IAC-1545 (*C. chinense*), 1579 (*C. annuum*) [244].

### 5.3. Aphids 

The cotton aphid (CA), *Aphis gossypii* and the green peach aphid (GPA), *Myzus persicae*, (*Hemiptera: Aphididae*), are the main species [245]. As direct pest, GPA causes chlorosis, leaf defoliation, flower, and fruit abortion and reduces photosynthesys. Moreover, GPA represents an efficient vector for many pepper destroying viruses including PepSMV, PepMoV and PepYMV [127].

Only a few studies to identify sources of resistance to GPA in *Capsicum* spp. have been published. Bosland and Ellington [246], found one *C. pubescens* accession showing antixenosis rather than antibiosis resistance to the GPA. However, no information has so far been reported on the use of this germplasm in *C. annuum* breeding for aphid resistance. 

Sun and colleagues [247], screened 74 pepper accessions, belonging to *C. annuum*, *C. frutescens*, *C. chinense* and *C. baccatum*, for resistance to GPA. The authors identified three *C. baccatum* accessions with high (PB2013071) or intermediate resistance (PB2013062 and PB2012022) and elucidated possible mechanisms of aphid resistance. The highly resistant genotype resulted in a severely reduced uptake of phloem, a significant callose deposition due to feeding of GPA, and in the accumulation of ROS (reactive oxygen species) [247].

Very recently, two major QTLs for resistance were detected and validated on pepper Chr 2 [248]. The analysis was carried out in an F_2_ population derived from the intraspecific cross between the highly resistant *C. baccatum* PB2013071 and the susceptible PB2013046. The identified QTLs *Rmprp-1* and *Rmpas-1* inhibited the reproduction and affected GPA survival, respectively. Moreover, *Rmprp-1* was located in a genomic region of 96 kb which is predicted to encode four analogs of resistance genes of the receptor-like kinase family containing a leucine-rich repeat domain (LRR-RLKs). Regarding CA, sources of resistance were found in *C. annuum* germplasm by the choice and non-choice tests [249]. The resistant accession IPB C20 made the shortest longevity and reproduction time of melon aphid compared to the other genotypes tested. Moreover, the same genotype IPB C20 was able to suppress aphid progenies.

### 5.4. Lepidopterous and Leaf Miner Pests

Cotton bollworm (*Helicoverpa armigera*) (*Lepidoptera: Noctuidae*) is the main moth that causes pepper damage. In Europe, the pest is of economic importance in Portugal and Spain and of lesser importance in other countries where it is also established. In 2003, *H. armigera* was a serious problem on pepper crops in Southern Italy (Metaponto area). Thirty percent of the pepper fruits and 70%–80% of the pepper plants were damaged. The larvae fed on leaves, flowers and fruits, with fruits recording the most serious damages [250] (Figure 4c). 

The analysis of thirty-three genotypes with different levels of damages caused by cotton bollworm under field conditions allowed to identify seven pepper genotypes (SL-37, Arka Lohith, Purired, Devarhippargi, TC-1, Button and H.C.-28) as resistant [251]. 

American serpentine leaf miner (*Liriomyza trifolii*) (*Diptera: Agromyzidae*) is a well-known pest with a broad host range among and leaf and fruit vegetable crops, attacking over 120 plant species. After hatching from the eggs, feed on the mesophyll tissues in the leaves and form serpentine mines, which can reduce significantly the photosynthetic activity of the plant. Resistance mechanisms against this pest were found in some inbred lines of *C. chinense* (G84, G110, and G37) [252]. Another source of resistance was detected in cv. Sakigake 2-go (*C. annuum* var. *angulosum*) [253].

### 5.5. Broad Mites 

The broad mite, *Polyphagotarsonemus latus* (*Acari*: T*arsonemidae*), is a polyphagous pest that attacks several important crops worldwide. This pest damages the outer cells of leaves as they feed on the plant sap. Leaves become distorted, bronze-colored, stiff, and rolled; flowers become distorted and fail to open normally; fruits are distorted and loss of yield is observed (Figure 4d). In extreme cases, plants are killed by the infestation. Resistant genotypes (Jwala, RHRC, Errect, and AGC-77) to *P. latus* were found [257] (Table 4); moreover, sources of resistance (Pant C-1; LCA-304 and LCA-312) both to *P. latus* and thrips *Scirtothrips dorsalis*, were identified [255]. More recently, Latha and Hunumanthraya [256], screening thirty-one chilli genotypes for thrips (*S. dorsalis*) and mite resistance under field condition, identified four *Capsicum* spp. accessions (DCC-3, DCC-185, DCC-109, and DCC-89) as moderately resistant to both pests. The authors highlighted that some morphological and biochemical characters (trichome density, chlorophyll, and phenol content) were negatively correlated with the population of thrips, mites and Leaf Curl Index.

### 5.6. Root-knot Nematodes

Root-knot nematodes (RKN) (*Nematoda*: *Meloidogyne*) belong to the genus *Meloidogyne* which includes 90 species, the most important of which in terms of damages and diffusion are *M. incognita*, *M. arenaria* and *M. javanica* (Figure 4e). Nematodes disease occurs in both open field and greenhouses, and prediction of the crop losses that a certain population density of nematode may cause is of importance to decide whether to cultivate pepper or not [272]. Another species that has gained importance recently is *M. enterolobii* for which, the sources of resistance against the major species of *Meloidogyne* are ineffective on its control [273]. Two genotypes, named UFGFR 05 (C. frutescens) and UFGCH 24 (*C. chinense*) are recently identified as resistant to *M. enterolobii* [269].

Resistance mechanisms to *M. arenaria* (races 1 and 2), *M. incognita* and *M. javanica* identified in *C. annuum*, *C. chinense*, *C. chacoense* and *C. frutescens* are conditioned by a single dominant gene designated *N* gene [2]. In *C. annuum*, resistance to RKN is also associated with several dominant genes (*Me* genes) that act independently in gene-for-gene interactions [262,268]. Six *Me* genes have previously been shown to be stable at high temperatures in three highly resistant and genetically distant accessions, PI322719, PI201234, and CM334. Some genes (*Me4, Mech1* and *Mech2*) are specific to certain *Meloidogyne* species or populations, whereas others (*Me1*, *Me3*, and *Me7*) are effective against a wide range of species, including *M. arenaria*, *M. javanica*, and *M. incognita*. 

However, the high genomic plasticity and genetic diversity exhibited by RKNs confer them a high potential to adapt to the host and an ability to develop virulent populations that break down the pepper plant resistance [274,275]. Nonetheless, a fitness cost associated with virulence has been observed and the joint management of diversified resistance sources together with adapted cultivation practices may well provide effective and sustainable control [276]. Particularly, two major *R* genes that differ in their mechanisms (*Me1* and *Me3*) into a single cultivar, seems the most secure and durable strategy after three years of experimentation [277].

*Me3* and *Me4* were found to be linked 10 cM each through BSA in a segregant population derived from the cross-Yolo Wonder (susceptible) X PM687 (resistant) [277]. These genes, along with *Mech1*, *Mech2*, *Me1* and *Me7* made the main cluster of 28 cM on Chr 9 [262]. Comparative mapping evidenced a colinearity with Chr 12 in both tomato and potato demonstrating the existence of orthologous regions for nematode resistance in Solanaceae. Crossing lines homozygous for *N* (Carolina Wonder and Charleston Belle) to lines homozygous for *Me3* (HDA 149 and PM 687) and employing allelism test, showed that the two genes were distinct [278]. A subsequent study found co-localization of *N*-gene in the *Me* genes cluster on the Chr 9, reporting the *N* gene allelic to *Me7* and located 7 cM apart from *Me1*, and 2 cM from *Me3* [261].

A genetic mapping study using F_2:3_ families derived from the cross Yolo Wonder × Doux Longd es Landes, allowed to identify a cluster on Chr 1 including three tightly linked QTLs with broad mechanisms of resistance against *M. incognita*, *M. arenaria*, *M. javanica*, respectively. A fourth QTL, providing specific resistance to *M. javanica* was mapped on Chr 9 [97]. 

Although several genes against root-knot have been identified, none of them has been cloned. Chen and collaborators [263], reported the first cloning study of *CaMi*, a candidate root-knot nematode resistance gene isolated from the resistant pepper line PR205. Transgenic tomato plants carrying the full coding genomic region of *CaMi* evidenced improved resistance against the root-knot nematodes compared to untransformed susceptible plants although not heritable. *CaMi* gene exerted a hypersensitive response (HR) as well as many necrotic cells around nematodes. Mao and colleagues [279], isolated and cloned from the resistant line HDA149, *CaRKNR*, an NBS-LRR gene showing homology to the tomato root–knot resistant gene Mi-1.2. The gene was mapped on Chr 6 and did not belong to *Me* family genes. In cloned plants, the expression level of *CaRKNR* increased up to four times while the silencing of the gene in HDA149 reduced the resistance to nematodes.

Different studies targeted at the development of markers linked to nematode-resistant gene for assisted breeding have been performed. Fazari and colleagues [261], developed PCR based markers tightly linked to *Me1*, *Me3*, *Me7* and *N* genes. A codominant CAPS marker located 1.13 cM away from the *Me1* gene, and a set of microsatellites tightly linked 0.8 cM away from the *N* gene have been also reported [280,281]. Finally, Wang and collaborators [282], fine mapped the region surrounding *Me1*, developing different PCR based markers closely linked. All these markers are useful for the marker-assisted breeding of nematode resistance in pepper.

## 6. Impact of Genomics and Future Challenges in Plant Disease Research

In recent years, a rapid increase in genomics has enabled the implementation of novel approaches toward the understanding of the molecular mechanisms underpinning resistance to pathogens. Next-generation sequencing technologies can be applied to provide whole-genome sequencing of pathogens and to develop high-throughput molecular markers for QTL mapping and gene discovery. Breeding programs are benefiting from these signs of progress in terms of precision and speediness to achieve results. Indeed, conventional molecular approaches are laborious and time-consuming. The QTL studies performed up to the early 2000s led to the development of genetic maps consisting of a few hundred markers to use for gene discovery and markers assisted selection. QTLs were often positioned in large intervals with difficulty in transferring them due to linkage drag. Moreover, markers were not always reliable due to recombination mechanisms leading to linkage breaking. As a result, many available molecular markers are not applicable in breeding for resistance. NGS-based genotyping produces instead thousands of single nucleotide polymorphism which allow detecting loci involved in the resistance to pathogens narrowing down the regions underlying genes of interest. Reduced representation sequencing method for genotyping such GBS (genotyping by sequencing) or RADseq (restriction site-associated DNA sequencing) are paramount, allowing high throughput genome scans at relatively low cost [93,168,283]. These NGS technologies, therefore, can be used to generate diagnostic markers able to detect the allelic variation within resistance genes. In addition to genomic-based breeding, NGS can be applied to unravel the diversity of the genome sequences of pathogen strains to identify specific virulence genes. Moreover, it can be applied to generate a large dataset of sequenced transcriptomes associated with pathogen virulence or to investigate the expression of effector proteins during the early stages of infection [284]. The future of NGS is shifting toward whole-genome sequencing, allowing to resolve key questions related to the function of virulence genes, the mechanism of resistance and the evolution of pathogens. The major accessibility to platforms as well the easiest analysis and management of data make the use of these technologies affordable to pathologists, geneticists, and breeders, covering different branches of research with the final target of better management and control of diseases.

## Figures and Tables

**Table 1:** Sources of resistance/tolerance to fungal and bacterial diseases in *Capsicum* spp.

Disease Name	Species	Sources of Resistance/Tolerance		
		Accessions/Lines/Genotypes	Species	References
**Fungal diseases**				
**Powdery mildew**	***Leveillula taurica***	H3, H-V-12 [H3’ x ‘Vania’ (susceptible)], 4638	*C. annuum*	[14]
		CNPH36, CNPH38, CNPH50, CNPH52, CNPH279, CNPH288, KC604, KC605, KC608	*C. baccatum*	[15,16]
		IHR 703	*C. frutescens*	[15]
		KC616	*C. chinense*	[16]
		KC638, KC640, KC641, KC642, KC643, KC644	*C. pubescens*	[16]
		PI 6440507	*n.a.*	[20]
***Phytophthora*** **root rot**	***Phytophthora capsici***	PI 201234	*C. annuum*	[21]
		PI 201232, PI 201237, PI 640532	*C. annuum*	[22]
		PBC137	*C. annuum*	[21]
		PBC602	*C. annuum*	[21]
		Serrano Criollo de Morelos (CM334)	*C. annuum*	[23]
		AC2258	*C. annuum*	[24]
		Perennial	*C. annuum*	[23]
		Grif 9073, PI 439297	*C. annuum*	[25]
		BG102, BG107	*C. annuum*	[26]
**Antrachnose fruit rot**	***Colletotrichum truncatum***	PBC80, PBC81, CA1422	*C. baccatum*	[27,28]
		PBC932, CO4714	*C. chinense*	[27,28]
	***Colletotrichum scovillei***	PBC80, PBC81	*C. baccatum*	[27,28]
		PRI95030	*C. chinense*	[29,30]
		UENF 1718, UENF 1797	*C. baccatum* var. *pendulum*	[31]
	***Colletotrichum siamense***	CO4714	*C. chinense*	[28]
		Jinda, Bangchang, 83–168	*C. annuum*	[28]
		Khee Noo, Karen	*C. frutescens*	[28]
	**Both *C. truncatum* and *C. siamense***	Acchar lanka, CA-4, Pant C-1, Punjab Lal, Bhut Jolokia, BS-35	*C. annuum*	[32]
***Verticillium*** **wilt**	***Verticillium dahliae***	Grif 9073, PI 281396, PI 281397, PI 438666, PI 439292, PI 439297, PI 555616, PI 594125	*C. annuum*	[25]
***Fusarium*** **wilt**	***Fusarium solani***	P3, JNA2 × ACB1 × 9608D, Rajaput × P3	*C. annuum*	[33]
	***Fusarium oxysporum f.sp. capsici***	Punjab Lal, Solan Red, Pachhad Yellow, Solan Yellow, Pant C-1	*C. annuum*	[34]
	***Fusarium verticilloides* and *F. pallidoroseum***	Masalawadi, SC-120, Phule C-5, SC-335, SC-415, SC-1 07, SC-348, SC-108, LCA-304 Arka Lohit, Pusa Jwala, Pant C-2	*C. annuum*	[35,36]
***Rhizoctonia*** **root rot**	***Rhizoctonia solani***	PI 439410, PI 5556119	*C. baccatum*	[37]
		Long Chili, PI 167061	*C. annuum*	[37]
**Bacterial diseases**				
**Bacterial leaf spot**	***Xanthomonas spp***	PI 260435	*C. chacoense*	[38,39,40,41,42,43,44]
		PI 235047	*C. pubescens*	
		PI 163192, PI 271322, Pep13, PI 163192	*C. annuum*	
		UNEF1556	*C. baccatum* var. *pendulum*	
**Bacterial wilt**	***Ralstonia solanacearum***	Perennial, Narval, MC4, CA8, PI 322719, LS2341, PM687, YCM334	*C. annuum*	[45,46,47]
		Heiser 6240, LS 2390	*C. frutescens*	[48]
		LS1716, PBC385, PBC066, BC204, PBC1347, CNPH143 (MC4), CNPH14 (MC5),CNPH145 (HC10)	*C. baccatum*	[48]

**Table 2:** List of mapping populations, genetic map and Quantitative Trait Loci (QTLs) for biotic stress resistance in pepper.

Disease Name	Species	Mapping Population	Resistant Parent	Susceptible Parent	Individuals	Linkage Map#	N° of Markers	N° of QTLs /[gene]	Chr Location*	Ref
Powdery Mild	*Leveillula taurica*	Double Haploid	*C. annuum* ‘H3’	*C. annuum* ‘Vania’	101	AFLP, RAPD, RFLP	134	*5*	5, 6, 9, 10, 12	[18]
Powdery Mild	*Leveillula taurica*	F_2:3_	*C. annuum* ‘VK515R’	*C. annuum* ‘VK515S’	102	SNPs	96	[*PMR*1]	4	[19]
Powdery Mild	*Leveillula taurica*	F_2_	*C. annuum* ‘PM Singang’	*C. annuum* ‘Bukang’	80					
Powdery Mild	*Leveillula taurica*	Patented	*C. annuum* PBC167 (PI640507)	*na*	*na*	*na*	*na*	*na*	1, 8	[49]
Powdery Mild	*Leveillula taurica*	BC_1_F_2_	*C. annuum* PBC167 (PI640507)	*C. annuum* SBY 99–1179	96	SNPs	*na*	*na*	4	[20]
*Phytophthora* root rot	*Phytophthora capsici*	Double Haploid	*C. annuum* “Vania with introgression from PI201234”	*C. annuum* ‘H3’	101	AFLP, RAPD, RFLP	135	13	3, 5, 7, 10, 11, 12	[61]
*Phytophthora* root rot	*Phytophthora capsici*	Double Haploid	*C. annuum* ‘Perennial’	*C. annuum* ‘Yolo wonder’	114	AFLP, RAPD, RFLP	154	11	2, 5, 10	[61]
*Phytophthora* root rot	*Phytophthora capsici*	F_2_	*C. annuum* ‘Criollo de morelos CM334’	*C. annuum* ‘Yolo wonder’	151	AFLP, RAPD, RFLP	64	20	1, 4, 5, 6, 11, 12	[61]
*Phytophthora* root rot	*Phytophthora capsici*	RIL	*C. annuum* ‘PI201234’	*C. annuum* ‘PSP-11’	*na*	AFLP, RAPD, SSR, SCAR	144	16	*na*	[62]
*Phytophthora* root rot	*Phytophthora capsici*	F_2_	*C. annuum* ‘CM334’	*C. annuum* ‘Joe E. Parker’	*na*	AFLP, RAPD, SSR, SCAR	113	5	*na*	[62]
*Phytophthora* root rot	*Phytophthora capsici*	Double Haploid	*C. annuum* ‘AC2258’	*C. annuum* ’K9-11’	176	AFLP, RAPD, RFLP, SCAR, CAPS	518	3	1, 5, 11	[65]
*Phytophthora* root rot	*Phytophthora capsici*	F_2_	*C. chinense ’*PI 159234’	*C. annuum* ‘Numex Rnaky’	75	RAPD, SCAR	300	1	5	[51]
*Phytophthora* root rot	*Phytophthora capsici*	F_2_	*C. annuum* ‘Criollo de morelos CM334’	*C. annuum* ‘Numex Rnaky’	94	RAPD, SCAR	300			
*Phytophthora* root rot	*Phytophthora capsici*	Double Haploid	*C. annuum* ‘Criollo de morelos CM334’	*C. annuum* ‘Manganji’	96	SSR	118	2	3, 5	[63]
*Phytophthora* root rot	*Phytophthora capsici*	F_2_	*C. annuum* ‘Criollo de morelos CM334’	*C. annuum* ‘Chilsungcho’	100	RFLP, SSR, WKRY	241	7	5, 6, 8, 9	[64]
*Phytophthora* root rot	*Phytophthora capsici*	RILs_F_8_	*C. annuum* ‘YCM334’	*C. annuum* ‘Tean’	126	AFLP, CAP, SSR	249	15	5, 10, 11	[66]
*Phytophthora* root rot	*Phytophthora capsici*	RILs_F_8_	*C. annuum* ‘YCM334’	*C. annuum* ‘Tean’	126	HRM	41	4	4, 5	[67]
*Phytophthora* root rot	*Phytophthora capsici*	RILs_F_6_	*C. annuum* ‘YCM334’	*C. annuum* ‘Early jalapeno’	63	SPP (single position polym)	3814	10	2, 3, 4, 5, 6	[70]
*Phytophthora* root rot	*Phytophthora capsici*	RILs_F_7_	*C. annuum* ‘YCM334’	*C. annuum* ‘Early jalapeno’	66	SNP array	3887	*CaDMR1*	5	[71]
*Phytophthora* root rot	*Phytophthora capsici*	Two BC_1_; one F_2_	*C. annuum* ‘Criollo de morelos CM334’	NMCA10399	222, 372; 259	SLAF seq	>40,000	*PhR10*	10	[72]
Antrachnose disease	*Colletotrichum gloeosporioides and C. capsici*	F_2_	*C. chinense* ’PRI95030’	*C. annuum* ‘Jatilaba’	346	AFLP, SSR	266	4	*na*	[29]
Antrachnose disease	*Colletotrichum acutatum*	F_2_	*C. baccatum* var. *pendulum*	*C.baccatum* ‘Golden-aji’	126	AFLP, SRAP, SSR	327	19	3, 4, 5, 6, 7, 8. 9	[79]
Antrachnose disease	*Colletotrichum acutatum*	BC_1_	*C. chinense* ’PBC932’	*C. annuum* ‘77013’	186	CAPS, INDEL, SSR	385	12	3, 5, 7, 10, 12	[80]
Bacterial Wilt	*Ralstonia solanacearum*	Double Haploid	*C. annuum* ’LS2341’	*C. annuum* ‘California wonder’	94	AFLP, SSR	359	1	1	[86]
Bacterial Wilt	*Ralstonia solanacearum*	Double Haploid	*C. annuum* PM687 (PI322719)	*C. annuum* ‘Yolo wonder’	117	AFLP	117	6	2, 4, 6, 9, 10, 11	[87]
Potyviruses	*PVY* and potyviruses	Double Haploid	*C. annuum* ‘Perennial’	*C. annuum* ‘Yolo wonder’	94	RAPD, RFLP	172	11	3, 4, 7, 9, 11	[88]
Potyviruses	*PVY*	Double Haploid	*C. annuum* ‘Perennial’	*C. annuum* ‘Yolo wonder’	350	AFLP, SNPs, SSCP, SSR	236	4	1, 3, 6, 9	[89]
*Cucumoviruses*	CMV	Double Haploid	*C. annuum* ‘Perennial’	*C. annuum* ‘Yolo wonder’	94	RAPD, RFLP	138	7	3, 11, 12	[88]
*Cucumoviruses*	CMV	F_3_ families	*C. annuum* ‘Perennial’	*C. annuum* ‘Maor’	180	AFLP, RAPD, RFLP	177	4	4, 6, 11	[90]
*Cucumoviruses*	CMV	Double Haploid	*C. annuum* ’Vania’	*C. annuum* ‘XJ0630’	101	AFLP, RAPD, RFLP	184	6	5, 11, 12	[91]
*Cucumoviruses*	CMV (HB)	F_2_ and BC	*C. annuum* “BJ0747”	*C. annuum* ‘H3’	334	ISSR, SSR	137	5	5, 7, 11	[92]
*Cucumoviruses*	CMV (P1)	F_3_	*C. annuum* ‘A1’	*C. annuum* ‘2602’	174	GBS	906	2	5, 10	[93]
*Cucumoviruses*	CMV (FNY)	F_2_	*C. frutescens* ‘PBC688’	*C. frutescens* ‘G29’	190	SLAF	36.847	1/*[CA02g19570]*	11/2	[94]
*Cucumoviruses*	CMV (HB-jz)	F_2_	*C. annuum* ‘BJ0747’	*C. annuum* ‘XJ0630’	195	SLAF	14,601	3	11, 12	[95]
Thrips	*Frankliniella occidentalis*	F_2_	*C. chinense* ‘4661’	*C. annuum* ‘AC 1979’	196	AFLP, SNP, SSR	171	1	6	[96]
Root-knot nematodes	*Meloidogyne incognita*, *M. arenaria*, *M. javanica*	F_2:3_	*C. annuum* ‘Yolo wonder’	*C. annuum* ‘Doux Longd es Landes’	130	SCAR, SNP, SSR	326	4	1, 9	[97]

**Table 3:** Sources of resistance/tolerance to virus diseases in *Capsicum* spp.

Species	Sources of Resistance/Tolerance		
	Accessions/Lines/Genotypes	Species	Reference
***Genus: Orthotospovirus***			
*Tomato spotted wilt orthotospovirus* (TSWV)	PI 152225, PI 159234, PI 159236, 7204, CNPH-275, AC09-207, 7204, PI -15, C00943, ECU-973	*C. chinense*	[133,140,142,201]
	PIM26-1, C-153	*C. baccatum*	[150]
	PI 264281	*C. annuum*	[201]
*Capsicum chlorosis orthotospovirus* (CaCV)	PI 90972	*C. chinense*	[151]
***Genus: Potyvirus***			
*Pepper mottle virus* (PepMoV)	Tabasco (CGN 21546)	*C. frutescens*	[202]
	Avelar, 9093	*C. annuum*	[163,203]
*Pepper yellow mosaic virus* (PepYMV)	UENF 1624, UENF 1732, UENF 1764, UENF 1770	*C. baccatum* var. *pendulum*	[204]
*Potato virus Y* (PVY)	Perennial (partially resistant)	*C. annuum*	[205]
	Pen 3.4, CGN 17015 (Amarjllo)	*C. baccatum*	[202]
*Potato virus Y* pathotype 0 (PVY-0); *Tobacco etch virus* (TEV); *Pepper mottle virus* (PepMoV)	PI 159236, PI 152225	*C. chinense*	[155]
*Potato virus Y* pathotype 0 (PVY-0)	Yolo Y	*C. annuum*	[157]
*Potato virus Y* pathotypes 0 and 1 (PVY-0-1); *Tobacco etch virus* (TEV)	PI 264281, SC46252, Florida VR2	*C. annuum*	[155]
*Potato virus Y* pathotypes 0, 1 and 2 (PVY-0-1); *Pepper mottle virus* (PepMoV)	CM334	*C. annuum*	[155,206]
*Tobacco etch virus* (TEV)	Agronomico 10C-5, Delray Bell, VR4	*C. annuum*	[202]
*Chilli veinal mottle virus* (ChiVMV)	Perennial	*C. annuum*	[205]
***Genus: Cucumovirus***			
*Cucumber mosaic virus* (CMV)	Perennial, Bukang, Lam32, Vania, Sapporo-oonaga, Nanbu-oonaga, BJ0747	*C. annuum*	[91,92,164,175,177,178]
	BG2814-6, Tabasco (CGN 21546), LS1839-2-4	*C. frutescens*	[202,207]
	PI 439381-1-3	*C. baccatum*	[178]
***Genus: Begomovirus***			
*Pepper leaf curl virus* (PepLCV)	BS-35, GKC-29, Bhut Jolokia,	*C. annuum*	[192]
*Chilli leaf curl virus* (ChiLCV)	DLS-Sel-10, WBC-Sel-5, PBC-142, PBC-535	*C. annuum*	[208]
*Pepper yellow mosaic virus* (PepYMV)	PP1037-7644-1, PBC148, PBC149, PBC502, PBC518, PBC601, PP99	*n.a.*	[194]
*Pepper huastego yellow vein virus* (PHYVV)	UAS12	*C.annuum*	[197]
*Pepper golden mosaic virus* (PepGMV)	BG-3821	*C. chinense*	[198]
***Genus: Tobamovirus***	PI 315008, PI 315023, PI 315024, PI 159236, PI 152225, KC667	*C. chinense*	[198,209]
	Bruinsma Wonder, Verbeterde Glas, KC780, Nanbu-Ohnaga	*C. annuum*	[209,210]
	Tabasco	*C. frutescens*	[209]
	PI 260429.	*C. chacoense*	[209]
	PI 439381-1-3	*C. baccatum*	[209]

**Table 4:** Sources of resistance to arthropod and nematode pests in *Capsicum* spp.

Disease name	Species	Sources of resistance/tolerance		
		Accessions/Lines/Genotypes	Species	References
**Insects**				
**The south east Asian pest thrips and Western flower thrips**	*Thrips parvispinus and Frankliniella occidentalis*	AC 1979, Bisbas, Keystone Resistant Giant, CM 331,	*C. annuum*	[254]
		1553, Aji Blanco Christal	*C. baccatum*	[254]
**Western flower thrips**	*Frankliniella occidentalis*	CPRO-1	*n.a.*	[238]
**Chilli thrips**	*Scirtothrips dorsalis*	Pant C-1, LCA-304, LCA-31	*C. annuum*	[255]
		DCC-3, DCC-185, DCC-109, DCC-89	*n.a.*	[256]
**Tobacco whitefly**	*Bemisia tabaci*	CM331, Seranno, California Wonder 300	*C. annuum*	[242]
		P2, P4, ACC1, ACC12	*n.a.*	[243]
		IAC-1544	*C. frutescens*	[244]
		IAC-1545	*C. chinense*	[244]
		IAC-1579	*C. annuum*	[245]
**Green peach aphid**	*Myzus persicae*	PB2013071, PB2013062, PB2012022	*C. baccatum*	[246]
**Cotton aphid**	*Aphis gossypii*	IPB C20	*C.annuum*	[249]
**Cotton bollworm**	*Heliotis armigera*	SL-37, Arka Lohith, Purired, Devarhippargi, TC-1, Button, H.C.-28	*n.a.*	[251]
**American serpentine leafminer**	*Liriomyza trifolii*	G84, G110, G37	*C. chinense*	[252]
		Sakigake 2-go	*C. annuum* var *angulosum*	[253]
**Broad mites**	*Polyphagotarsonemus latus*	Jwala, RHRC, Errect, AGC-77 ,	*C. annuum*	[257]
		Pant C-1, LCA-304, LCA-31	*C. annuum*	[255]
		DCC-3, DCC-185, DCC-109, DCC-89	*n.a.*	[256]
**Nematodes**				
**Root-knot nematodes**	*Meloidogyne incognita*	528-8, 529-8, 46-530/7	*C. chacoense*	[258,259]
		PA-353, PA-398, PA-426, 201-26, 547-7, 56-547/7	*C. chinense*	[258,260]
		586-12, 28-201, Santanka XS, White Kandhari	*C. frutescens*	[259,261]
		Pusa Jwala, Carolina Cayenne, PM687, PM217, PR205, PM702	*C. annuum*	[262,263,264,265,266]
**Peanut root-knot nematode**	*Meloidogyne arenaria*	530-8, 213-8	*C. chacoense*	[258]
		201-8, 550-10, 559-18, 546-6, PA-353, PA-398, PA-426	*C. chinense*	[258,267]
		589-20, Santanka XS	*C. frutescens*	[258,260]
		PM217, PM687, PM702	*C. annuum*	[262]
**Sugarcane eelworm**	*Meloidogyne javanica*	530-8	*C. chacoense*	[258]
		201-16, 201-21, 550-10, PA-353, PA-398, PA-426	*C. chinense*	[258,267]
		589-20, Santanka XS	*C. frutescens*	[258,268]
		PM217, PM687, PM702	*C. annuum*	[262]
**Pacara earpod tree root-knot nematode**	*Meloidogyne enterolobii*	UENF 1730, UFGCH 24	*C. chinense*	[269,270]
		UFGFR 05	*C. frutescens*	[269]
**Northern root-knot nematode**	*Meloidogyne hapla*	PI 441641, 201-16, 201-21	*C. chinense*	[259,271]
		PI 439381, PI267729	*C. baccatum*	[271]
		589-20	*C. frutescens*	[258]
**Columbia root-knot nematode**	*Meloidogyne chitwoodi*	PM217, PM702	*C. annuum*	[262]

## Abbreviations

QTL	Quantitative Trait Locus
NBS-LRR	Nucleotide-binding site leucine-rich repeat
SCAR	Sequence Characterized Amplified Region
SNP	Single Nucleotide Polymorphysms
MAS	Marker-assisted selection
GBS	Genotyping by sequencing
RAPD	Random amplification of Polymorphic DNA
RFLP	Restriction Fragment Length Polymorphism
cM	Centimorgans
RIL	Recombinant inbred lines
AFLP	Amplified Fragment Length Polymorphism
BSA	Bulked Segregant Analysis
*Xs.*	*Xanthomonas*
KASP	Kompetitive Allele-Specific PCR
BW	Bacterial wilt
TSWV	*Tomato spotted wilt orthotospovirus*
CaCV	*Capsicum chlorosis orthotospovirus*
PVY	Potyviruses
TEV	*Tobacco etch virus*
PepMoV	*Pepper mottle virus*
TMV	*Tobacco mosaic virus*
ToMV	*Tomato mosaic virus*
CMV	*Cucumber mosaic virus*
